# Colonic resection or self-expanding metal stents for obstructive left colon cancer: results of a national multicenter prospective cohort study (CROSCO-1)

**DOI:** 10.1007/s00464-026-12929-9

**Published:** 2026-06-03

**Authors:** Alessio Giordano, Manuela Mastronardi, Paola Fugazzola, Giulia Montori, Ferdinando Agresta, Jacopo Martellucci, Fausto Catena, Federico Coccolini, Gabriele Anania, Gianluca Costa, Vincenzo Bottino, Nicola Cillara, Paolo Prosperi, Carlo Bergamini, Mauro Podda, The CROSCO-1 Collaborative Group, Marco Scatizzi, Luigi Ricciardielli, Diego Cuccurullo, Mario Guerrieri, Alberto Sartori, Massimo Sartelli, Luca Ansaloni, Nereo Vettoretto, Monica Ortenzi, Emanuele Botteri, Jacopo Martellucci, Mauro Marzano, Biagio Casagranda, Silvia Palmisano, Alan Biloslavo, Letizia Cecchini, Nicolò de Manzini, Pietro Fransvea, Silvia Tedesco, Caterina Puccioni, Ruggero Bollino, Massimiliano Casadei, Denise Marchesini, Mauro Podda, Lisa Seu, Federica Frongia, Anna Guariniello, Nicola Albertini, Claudia Di Leo, Andrea Borasi, Andrea Barberis, Francesco Ghiglione, Michele Tarricone, Maddalena De Capua, Mauro Ghirardi, Leo Licari, Gianluca Gambino, Vincenzo Sorce, Andrea Celotti, Martina Bonafede, Luca Mattia Quarti, Marco Clementi, Danilo Meloni, Irene Tucceri Cimini, Giorgio Ammerata, Michele Mazza, Giuseppe Sena, Giuseppe Palomba, Giovanni Aprea, Ciro de Martino, Giuseppe Miranda, Enrico Erdas, Gian Luigi Canu, Federico Cappellacci, Manish Kumar Agrawal, Akshay Anand Abhinav, Arun Sonkar, Amit Gupta, Deepak Rajput, Avijit Mondal, Jacopo Andreuccetti, Giusto Pignata, Claudia Zaghi, Franco Poli, Houshang Kalamian, Marco Milone, Anna D’amore, Giovanni De Palma, Giuseppe Curro, Michele Ammendola, Silvia Curcio, Andrea-Pierre Luzzi, Salvatore Carrabetta, Raquel Diaz, Valerio Caracino, Paola Salusso, Diletta Frazzini, Paolo Di Mattia, Chiara Toscano, Debora Maria Di Dio, Valeria Tonini, Lodovico Sartarelli, Maurizio Cervellera, Giuseppe Evola, Luigi Piazza, Lorenzo Ripamonti, Nicolò Tamini, Giulia De Carlo, Francesco Feroci, Carla Vaccaro, Benedetta Pesi, Antonello Mirabella, Stefano Mandalà, Dario Iadicola, Pasquale Cianci, Ivana Conversano, Fabio Curci, Paolo Ialongo, Pisicchio Simona, Rrozhani Valentina, Angelo Benevento, Maria Francesca Chiappetta, Federica Galli, Luigi Boccia, Giacomo Brentegani, Roberto Dusi, Raffaele Galleano, Omar Ghazouani, Elena Gambino, Massimo Calistri, Claudia Paolini, Luca Domenico Bonomo, Chiara Marafante, Antonella Nicotera, Paolo Pietro Bianchi, Giampaolo Formisano, Adelona Salaj, Fabio Staderini, Laura Fortuna, Damiano Bisogni, Rita Laforgia, Angela Pezzolla, Pierluigi Lobascio, Andrea Coratti, Giuseppe Giuliani, Francesco Guerra, Michele Marini, Costanzo Antonio, Michela Caprioli, Christian Cotsoglou, Beatrice Torre, Stefano Granieri

**Affiliations:** 1 Emergency Surgery Unit, Careggi University Hospital, Largo Brambilla 3, 50134 Florence, Italy; 2 General Surgery Unit, Department of Medicine, Surgery and Health Sciences, University Hospital of Trieste, Trieste, Italy; 3 Department of General Surgery I, University of Pavia, IRCCS San Matteo Hospital, Pavia, Italy; 4 Department of General Surgery, Ulss2 Marca Trevigiana, Vittorio Veneto, Treviso, Italy; 5 Emergency and Trauma Surgery Unit, Bufalini Hospital, Cesena, Italy; 6 Emergency and General Surgery Unit, Pisa University Hospital, Pisa, Italy; 7 Department of Surgery, Sant’Anna University Hospital, Ferrara, Italy; 8 Colo-Rectal Surgery Unit, Link Campus University, Rome, Italy; 9 Department of General and Laparoscopic Surgery, Bariatric and Metabolic Surgery Unit, Ospedale Evangelico Betania, Naples, Italy; 10 General Surgery Unit, P.O. Santissima Trinità, Cagliari, Italy; 11 Department of Surgical Science, Cagliari State University, Cagliari, Italy

**Keywords:** Colon cancer, Obstructive left colon cancer, Self-expanding metal stent (SEMS), Emergency surgery, Quality of life, Stoma rate

## Abstract

**Background:**

The optimal management of acute left-sided malignant colonic obstruction remains controversial. Emergency surgery (ES) is associated with substantial morbidity and high stoma rates, whereas self-expanding metal stents (SEMS) as a bridge to surgery (BtS) may convert an emergency into an elective setting. This study aimed to compare the safety, efficacy, and quality-of-life outcomes of ES versus SEMS/BtS.

**Methods:**

CROSCO-1 is a national, multicenter, prospective observational cohort study conducted in Italy (ClinicalTrials.gov NCT05801211). Consecutive adults with obstructive, non-metastatic left-sided colon cancer (June 2023–October 2024) were included. Patients underwent ES or SEMS followed by elective resection. The primary endpoint was 1-year stoma rate. Secondary outcomes included morbidity, mortality, length of stay, time to chemotherapy, readmissions, oncologic outcomes, and 1-year quality of life (EQ-5D-5L).

**Results:**

A total of 216 patients were analyzed (ES 144; SEMS/BtS 72). One-year follow-up for the primary outcome was available for 134 ESG and 70 SEMS/BtS patients. SEMS failure occurred in 8.3%. Major morbidity and mortality at 30 and 90 days were similar. The 1-year stoma rate was significantly lower after SEMS/BtS (44.4 vs 73.4%). SEMS/BtS reduced the risk of stoma persistence (adjusted OR 3.74 for ES; *p* = 0.016). SEMS/BtS was associated with fewer 30-day readmissions (4.2 vs 15.9%) and earlier chemotherapy initiation (76.1 vs 55.8%; *p* = 0.003). One-year oncologic outcomes were comparable, although limited by short follow-up. Quality of life was significantly better in the SEMS/BtS group.

**Conclusions:**

In selected patients, SEMS as a bridge to surgery is associated with lower 1-year stoma rates and improved quality of life without increasing short-term morbidity. These findings support a tailored, multidisciplinary approach in experienced centers.

**Supplementary Information:**

The online version contains supplementary material available at 10.1007/s00464-026-12929-9.

Colorectal cancer (CRC) is one of the most common cancers worldwide, with 1.4 million new cases per year. The cumulative lifetime risk of developing CRC is approximately 6% [1]. Large bowel obstruction is a common complication of advanced CRC, occurring in 8–13% of patients and representing the initial presentation in 10–30% of cases. In most cases, the left colon is involved [2]. Currently, with the increasing adoption of laparoscopic and advanced endoscopic techniques, the optimal management of this acute condition remains a matter of debate. Surgical options include primary tumor resection in the emergency setting with an immediate colorectal anastomosis (possibly associated with a diverting loop ileostomy) or a Hartmann’s procedure. The latter operation, although very popular, has shown a high morbidity rate, and in up to 40% of cases, the stoma becomes permanent [3].

Therefore, since the mid-1990s, endoscopically applied self-expanding metallic stents (SEMS) have been utilized as an alternative to surgery. Endoscopic stent placement was initially introduced for the palliative management of malignant colonic obstruction [4] and was subsequently extended to preoperative decompression as a bridge to surgery, as well as to definitive palliative treatment, with promising early results. In selected patients, self-expanding metal stents (SEMS) have been shown to effectively relieve intestinal obstruction in the short term, allowing time for optimization prior to elective colorectal resection. The primary aim of SEMS placement in obstructed colon cancer is to convert an emergency surgical scenario into an elective setting, restore bowel function, and potentially reduce morbidity, mortality, and the need for a stoma. Several randomized controlled trials (RCTs) and case-matched studies have reported controversial results and expressed concern regarding the effect of colonic stenting on short-term complications and long-term survival in patients with potentially curable disease, due to the potential risk of local advancement of the cancer and metastatic spread [5–9]. The aim of this study was to compare colonic stenting as a bridge to surgery with emergency surgery (including colorectal resection with primary anastomosis or Hartmann’s procedure) in the management of malignant left-sided large bowel obstruction, evaluating the safety and efficacy of the two strategies, as well as their impact on patients’ quality of life.

## Materials and methods

The CROSCO-1 study is a national, multicenter, prospective observational cohort study conducted in Italy. The study enrolled consecutive adult patients presenting acutely with obstructive left-sided colon cancer between June 2023 and October 2024, with a planned follow-up of 12 months. The study was registered in ClinicalTrials.gov (NCT05801211), approved by the Ethics Committee of the Central Area of the Tuscany Region (24040_oss) and endorsed by three national surgical societies (ACOI, Italian Association of Hospital Surgeons; SICE, Italian Society of Endoscopic Surgery and New Technologies; WSESit, World Society of Emergency Surgery Italian Chapter).

Eligible patients were adults (≥ 18 years) admitted on an urgent or emergency basis with clinical and radiological evidence of left-sided malignant colonic obstruction involving the descending colon, sigmoid colon, or rectosigmoid junction. The diagnosis was confirmed by a contrast-enhanced abdominal CT scan. Only patients without distant metastases or peritoneal carcinomatosis and who were fit for surgery were included. Histological confirmation of colonic adenocarcinoma was required.

Exclusion criteria were perforation, abscess, or fistula at presentation, pregnancy, and palliative treatment for metastatic or locally advanced disease.

Patients were managed according to routine clinical practice and local availability of endoscopic services. Based on the initial management strategy, patients were allocated to one of two groups: the Emergency Surgery Group (ESG), including patients undergoing emergency colorectal resection (Hartmann’s procedure or primary anastomosis with or without diverting stoma), and the SEMS/BtS group, including patients undergoing endoscopic placement of a self-expanding metal stent (SEMS) as a bridge to surgery followed by elective resection.

The choice of treatment was left to the attending surgical team and was not influenced by the study protocol.

Treatment allocation was therefore based on local availability of SEMS, endoscopic expertise, and clinical judgment. As a result, patients were not randomly assigned, and differences in baseline characteristics or clinical condition may have influenced treatment selection. Clinical outcomes were analyzed according to an intention-to-treat principle based on the initial treatment strategy.

Data were prospectively collected using a standardized electronic case report form hosted on the REDCap platform hosted by the Careggi University Hospital of Florence. Collected variables included demographic characteristics, comorbidities, perioperative data, postoperative complications, histopathological findings, stoma status, chemotherapy timing, oncological outcomes, and quality of life. Postoperative follow-up was conducted at discharge, 30 days, 90 days, and 12 months after surgery, primarily via telephone interview. Postoperative complications were graded according to the Clavien–Dindo classification. Quality of life at 1 year was assessed using the EQ-5D-5L questionnaire [10].

The primary outcome was the stoma rate at 1 year after tumor resection. Secondary outcomes included 30-day and 90-day major morbidity and mortality, length of hospital stay (LOS), time to initiation of adjuvant chemotherapy, stoma reversal rate and timing, one-year quality of life, one-year oncological outcomes (local recurrence, distant metastases, and mortality) [11].

## Statistical analysis

Continuous variables were reported as mean ± standard deviation or median with interquartile range (IQR), according to data distribution. Categorical variables were expressed as absolute numbers and percentages. Comparisons between groups were performed using the Mann–Whitney *U* test for continuous variables and the Pearson chi-square test or Fisher’s exact test for categorical variables, as appropriate. To explore factors associated with stoma presence at one year, univariate logistic regression analyses were performed. Variables with clinical relevance and/or showing association at univariate analysis with a *p* value lower than 0.10 were then entered into multivariable logistic regression models. Results were reported as odds ratios (OR) with 95% confidence intervals (CI). Model performance was assessed using the omnibus test of model coefficients and Nagelkerke’s R^2^. A two-sided *p* value < 0.05 was considered statistically significant. Statistical analyses were performed using SPSS Statistics version 24.0 (IBM, Armonk, NY, USA).

All analyses were conducted according to an intention-to-treat principle based on the initial treatment strategy. Patients with failed SEMS placement were retained in the SEMS group, and crossover cases were analyzed according to the initial intended treatment. For the primary outcome, analyses were restricted to patients with available 1-year follow-up data. Missing data were not imputed, and analyses were performed on available cases. Consequently, the number of patients included varied across specific analyses depending on data completeness. The corresponding denominators for each analysis are reported in the tables.

## Results

**Fig. 1:**
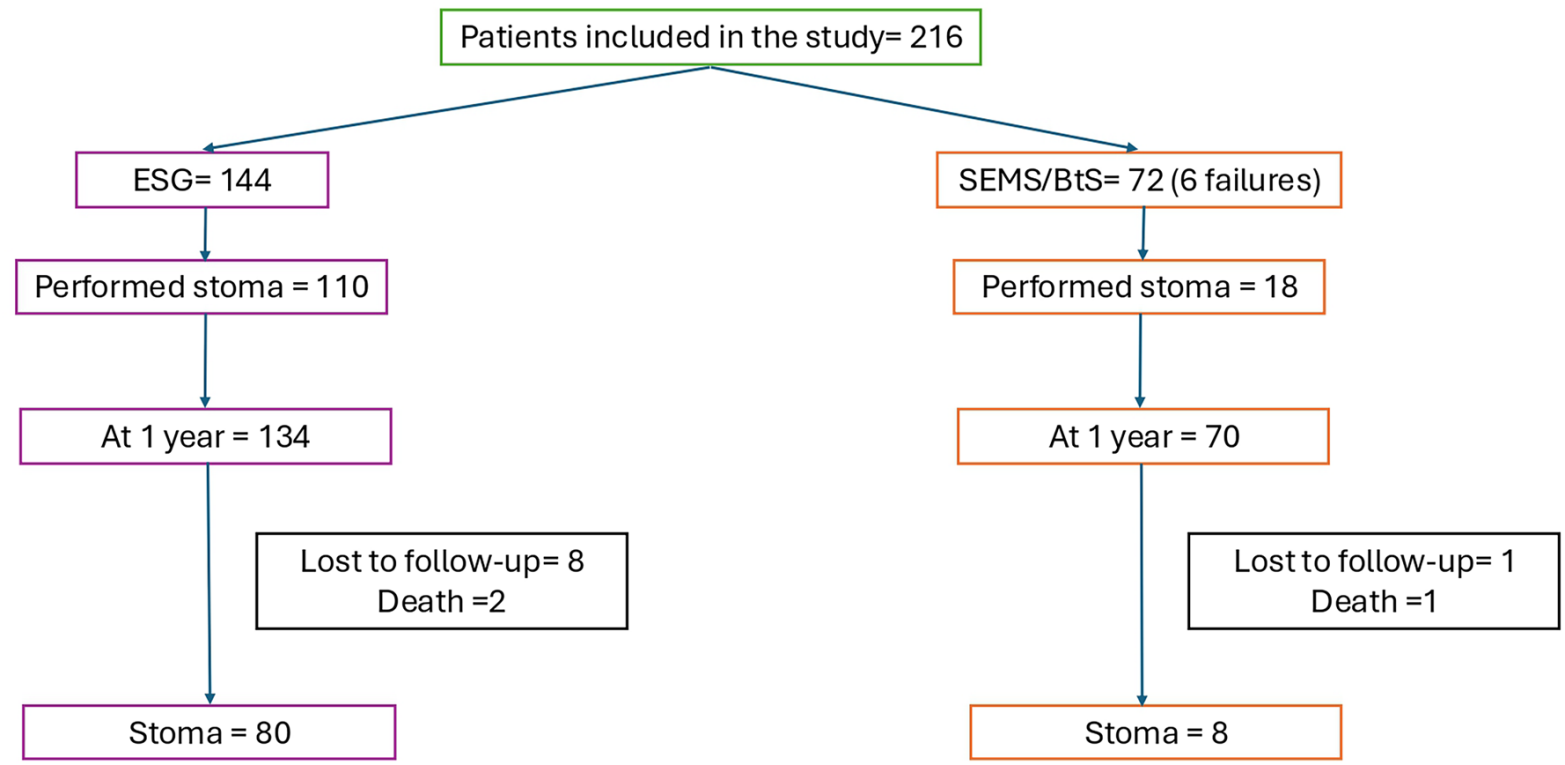
Flowchart of patient selection. *ESG* emergency surgery group; *SEMS/BtS* self-expanding metal stent/bridge to surgery

A total of 216 patients were included in the analysis (Fig. 1): 144 patients (66.7%) underwent ESG (29 colorectal resections with primary anastomosis, 34 colorectal resections with primary anastomosis with stoma protection, 81 Hartmann’s procedures), and 72 patients (33.3%) were managed with SEMS/BtS. Baseline demographic and clinical characteristics were largely comparable between groups, although patients in the SEMS/BtS group had a significantly higher BMI (SEMS/BtS = 27.0 ± 4.1 vs. ESG = 25.2 ± 5.4 kg/m^2^; *p* = 0.007). Mean age was 71.7 ± 13.9 years in the ESG and 68.9 ± 11.0 years in the SEMS/BtS group (*p* = 0.139). Sex distribution did not differ significantly between groups (male sex: 54.2 vs. 55.6%; *p* = 0.847). No significant differences were observed in American Society of Anesthesiologists (ASA) score distribution, Charlson comorbidity index (CCI), or prevalence of major comorbidities (Table 1). SEMS placement was technically successful in most patients, with a failure rate of 8.3% (six cases) and no reported perforations. Patients with SEMS failure or stent-related complications required surgical intervention, including five Hartmann’s procedures and one colorectal resection with primary anastomosis without stoma.

**Table 1:** Baseline characteristics of patients treated surgically or endoscopically

Variable	ESG (*n* = 144)	SEMS/Bts (*n* = 72)	*p* value
Age	71.65 ± 13.88	68.86 ± 10.97	0.139
Sex			0.847
Male	78 (54.2%)	40 (55.6%)	
Female	66 (45.8%)	32 (44.4%)	
BMI	25.20 (5.37)	27.00 (4.08)	0.007
CCI	5 (4)	5 (3)	0.319
ASA score			0.280
ASA 1	9 (6.3%)	4 (5.6%)	
ASA 2	47 (32.6%)	31 (43.1%)	
ASA 3	70 (48.6%)	33 (45.8%)	
ASA 4	18 (12.5%)	4 (5.6%)	
Type 2 diabetes			0.748
No	101 (70.1%)	54 (75.0%)	
DM with organ dysfunction	41 (28.5%)	17 (23.6%)	
DM without organ dysfunction	2 (1.4%)	1 (1.4%)	
Smoke habit			0.351
Never	83 (57.6%)	38 (52.8%)	
Current (1)	22 (15.3%)	18 (25.0%)	
Stopped < 6w (2)	3 (2.1%)	3 (4.2%)	
Stopped 6w–1y (3)	3 (2.1%)	1 (1.4%)	
Stopped > 1y (4)	33 (22.9%)	12 (16.7%)	
Liver disease	4 (2.8%)	1 (1.4%)	0.667
Other solid tumor	21 (14.6%)	13 (18.1%)	0.509
CKD: (under medication)	11 (7.6%)	7 (9.7%)	0.602
Previous abdominal surgery	68 (47.2%)	32 (44.4%)	0.700
Immunosuppressor therapy at admission	2 (1.4%)	3 (4.2%)	0.336
Other imaging in addition to CT scan	99 pts	44 pts	0.344
Abdominal X-ray (1)	67 (67.7%)	35 (79.5%)	
Ultrasound (2)	13 (13.1%)	4 (9.1%)	
Other (3)	19 (19.2%)	5 (11.4%)	
Endoscopic investigation (non operative)	64 (44.4%)	34 (47.2%)	0.634
Tumor location			0.793
Sigmoid (1)	67 (46.5%)	36 (50.0%)	
Discending colon (2)	39 (27.1%)	20 (27.8%)	
Recto-sigmoid (3)	38 (26.4%)	16 (22.2%)	
Operative timing			0.848
Expedited < 24 h (1)	71 (49.3%)	33 (45.8%)	
Immediate (2)	15 (10.4%)	9 (12.5%)	
Urgent < 12 h (3)	58 (40.3%)	30 (41.7%)	

Data are presented as mean ± standard deviation, median (interquartile range), or number (percentage), as appropriate

*EGS* emergency surgery group, *SEMS/Bts* self-expanding metal stents (SEMS) as a bridge to surgery (BtS), *BMI* body mass index, *CCI* Charlson comorbidity index, *ASA score* American society of Anesthesiologists score, *DM* diabetes mellitus, *CKD* chronic kidney disease

In the SEMS/BtS group, elective colorectal resection was performed after a mean interval of 17.02 ± 10.77 days from the initial emergency presentation.

In the ESG, emergency resection was performed predominantly via an open approach, whereas minimally invasive techniques were more frequently used in the SEMS/BtS group during elective surgery. The stoma rate was 76.4% (110/144) in ESG versus 25.0% (18/72) in SEMS/BtS (Table 2). Major postoperative complications (Clavien–Dindo ≥ III) at 30 and 90 days did not differ significantly between groups. Overall postoperative mortality at 30 and 90 days was comparable.

**Table 2:** Surgical approach and type of intervention performed between the two groups in the two different emergency and elective settings

	ESG (*n* = 144)	SEMS/BtS group (*n* = 72)	*p* value
Surgical approach			< 0.001
Laparoscopic (LPS)	25 (17.4%)	48 (66.7%)	
LPS → open	17 (11.8%)	2 (2.8%)	
Robotic	3 (2.1%)	14 (19.4%)	
Open	99 (68.8%)	7 (9.7%)	
Robotic → open	0 (0%)	1 (1.4%)	
Type of surgery			< 0.001
Hartmann’s procedure	81 (56.3%)	9 (12.5%)	
Left hemicolectomy + anastomosis + diverting stoma	29 (20.1%)	9 (12.5%)	
Left hemicolectomy + anastomosis without diverting stoma	34 (23.6%)	54 (75.0%)	

Data are presented as number (percentage)

*ESG* emergency surgery group, *SEMS/Bts* self-expanding metal stents (SEMS) as a bridge to surgery (BtS)

The length of postoperative hospital stay was shorter in the SEMS/BtS group compared with the ESG group (ESG 9 (IQR 5) days, SEMS/BtS group 4 (IQR 6.5) days, *p* value 0.112). The number of patients included in each analysis varied depending on data availability and completeness of follow-up. Therefore, denominators differ across outcomes and are reported in the corresponding tables. Stoma rate at 1 year was significantly higher in the ESG group (80/109, 73.4%) compared with the SEMS/BtS group (8/18, 44.4%; *p* = 0.025).

A significantly higher proportion of patients in the SEMS/BtS group started adjuvant chemotherapy within 90 days compared with the ESG group (76.1%, 54/71 vs 55.8%, 77/138; *p* = 0.003).

No significant differences were observed between groups in terms of early postoperative complications. However, the ESG showed a significantly higher rate of readmission within 30 days compared with the SEMS/BtS group (22/138, 15.9% vs 3/71, 4.2%; *p* = 0.013). The rate of reoperation within 30 days did not differ significantly between groups (6/138, 4.3% vs 0/71, 0.0%; *p* = 0.098). At 1-year follow-up, rates of distant metastases, local recurrence, and overall mortality were comparable between groups (Tables 3, 4; Supplementary Table 1).

**Table 3:** Postoperative outcomes, pathological findings, and time-related results according to treatment group

	ESG (*n* = 144)	SEMS/BtS (*n* = 72)	*p* value
Any complications	30 (20.8%)	9 (12.5%)	0.188
Highest post-operative Clavien–Dindo complication grade			0.546
Grade IIIa	11 (7.6%)	6 (8.3%)	
Grade IIIb	8 (5.6%)	1 (1.4%)	
Grade Iva	3 (2.1%)	1 (1.4%)	
Grade IVb	2 (1.4%)	0 (0.0%)	
Grade V	6 (4.2%)	1 (1.4%)	
Type of tumor histology			0.186
Adenocarcinoma	139 (96.5%)	66 (91.7%)	
Mucinous adenocarcinoma	5 (3.5%)	6 (8.3%)	
pT stage			0.592
pT1	1 (0.7%)	0 (0.0%)	
pT2	17 (11.8%)	9 (12.5%)	
pT3	99 (68.8%)	54 (75.0%)	
pT4	27 (18.8%)	9 (12.5%)	
pN stage			0.792
pN0	57 (39.6%)	25 (34.7%)	
pN1	66 (45.8%)	36 (50.0%)	
pN2	20 (13.9%)	11 (15.3%)	
pNX	1 (0.7%)	0 (0.0%)	
TNM stage			0.541
Stage I	9 (6.3%)	2 (2.8%)	
Stage II	60 (41.7%)	32 (44.4%)	
Stage III	75 (52.1%)	38 (52.8%)	
LOS from procedure to discharge	9 (5)	4 (6.5)	0.112
Days to stoma reversal	64.5 (35)	157 (138.5)	0.016

Data are presented as mean ± standard deviation, median (interquartile range), or number (percentage), as appropriate

*EGS* emergency surgery group, *SEMS/BtS* self-expanding metal stents (SEMS) as a bridge to surgery (BtS), *LOS* length of stay

**Table 4:** Short- and mid-term follow-up outcomes according to treatment group

Variable	ESG = 144	SEMS/BtS = 72	*p* value
Readmission within 30 days	22/138 (15.9%)	3/71 (4.2%)	0.013
Reoperation within 30 days	6/138 (4.3%)	0/71 (0.0%)	0.098
Highest Clavien–Dindo grade = V	1/138 (0.72%)	1/71 (1.4%)	1.000
Readmission within 90 days	10/138 (7.2%)	4/71 (5.6%)	0.776
Reoperation within 90 days	1/138 (0.7%)	0/71 (0.0%)	1.000
Started chemotherapy (within 90 days)	77/138 (55.8%)	54/71 (76.1%)	0.003
Metastases at 1 year	28/136 (20.6%)	9/71 (12.9%)	0.186
Local recurrence at 1 year	10/136 (7.3%)	3/71 (4.2%)	0.549
Death at 1 year	2/136 (1.5%)	1/71 (1.4%)	1.000
Stoma rate at 1 year	80/109 (73.4%)	8/18 (44.4%)	0.025
Stoma reversed within 90 days	5/110 (4.5%)	1/18 (5.6%)	1.000
Stoma reversed within 1 year	26/109 (23.9%)	5/18 (27.8%)	0.750

Data are presented as number (percentage)

Denominators vary across outcomes due to missing data and differences in follow-up availability. Analyses were performed on available cases for each specific endpoint

*ESG* emergency surgery group, *SEMS/BtS* self-expanding metal stents (SEMS) as a bridge to surgery (BtS)

Quality-of-life data at 1 year were available for 134 patients in the ESG and 70 patients in the SEMS/BtS group, corresponding to response rates of 98.5 and 97.2% among patients with available follow-up, respectively. Missing data were due to non-completion of the EQ-5D-5L questionnaire. Analyses were performed on available cases, and the corresponding denominators are reported in the tables. Across all five EQ-5D domains, patients treated with SEMS/BtS reported significantly better quality-of-life outcomes compared with those in the ESG. In the mobility domain, a higher proportion of SEMS/BtS patients reported no mobility problems (78.6 vs 58.2%), whereas moderate or severe limitations were more frequent in the ESG group (*p* = 0.049). Similarly, for self-care, a significantly larger proportion of SEMS/BtS-treated patients reported no difficulties with washing or dressing (82.9 vs. 53.0%), whereas moderate to severe self-care limitations were more common in the ESG (*p* < 0.001). Regarding usual activities, patients in the SEMS/BtS group more frequently reported no limitations (78.6 vs. 41.0%), while moderate or severe impairments were predominantly observed among ESG patients (*p* < 0.001). In the pain/discomfort domain, the absence of pain was reported by 68.6% of patients in the SEMS/BtS compared with 39.6% in the ESG, with higher rates of mild-to-moderate pain among ESG patients (*p* = 0.003). Finally, in the anxiety/depression domain, SEMS/BtS patients more frequently reported not being anxious or depressed (62.9 vs. 26.1%), whereas moderate to severe anxiety or depressive symptoms were more common in the ESG (*p* < 0.001) (Supplementary Table 2).

### Univariate and multivariable predictors of stoma presence at 1 year

Univariate and multivariable analyses were performed on patients with complete data for all variables included in the model (*N* = 127). Univariable logistic regression showed that increasing age was significantly associated with higher odds of having a stoma at 1 year (OR 1.043 per 1-year increase, 95% CI 1.013–1.073; *p* = 0.004). The ESG approach was also significantly associated with stoma persistence at 1 year (OR 3.448, 95% CI 1.241–9.583; *p* = 0.018). Similarly, a higher Charlson Comorbidity Index was associated with increased odds of stoma presence at 1 year (OR 1.185 per point, 95% CI 1.022–1.374; *p* = 0.025).

Sex, BMI, and postoperative complications were not significantly associated with stoma at 1 year (all *p* > 0.05). ASA grade was not significant overall, although patients with ASA II (vs ASA IV) had lower odds of stoma persistence (OR 0.200, 95% CI 0.040–0.996; *p* = 0.049) (Supplementary Table 3).

In the multivariable logistic regression model, surgical treatment remained independently associated with higher odds of stoma presence at 1 year (adjusted OR 3.740, 95% CI 1.278–10.943; *p* = 0.016). In contrast, age (adjusted OR 1.032, 95% CI 0.992–1.073; *p* = 0.117) and Charlson Comorbidity Index (adjusted OR 1.084, 95% CI 0.888–1.324; *p* = 0.429) were not significantly associated with stoma persistence after adjustment. The omnibus test indicated overall model significance (*χ*^2^ = 15.295; *p* = 0.002) (Table 5).

**Table 5:** Multivariable logistic regression for stoma presence at 1 year

Predictor	Adjusted OR	95% CI	*p* value
Surgical (vs endoscopic)	3.740	1.278–10.943	0.016
Age (per 1-year increase)	1.032	0.992–1.073	0.117
Charlson Comorbidity Index (per 1-point increase)	1.084	0.888–1.324	0.429

*OR* odds ratio, *CI* confidence interval

## Discussion

The optimal management of acute left-sided malignant colonic obstruction remains one of the most debated topics in colorectal surgery. Despite advances in perioperative care, emergency resection continues to be associated with substantial morbidity, mortality, and, in up to 40% of cases, permanent stoma formation [12, 13]. For short-term outcomes of stent implantation, Arezzo et al.’s systematic review [3], based on eight randomized controlled trials including 497 patients, showed that stenting combined with elective surgery significantly reduced the rate of immediate stoma (34 vs. 51%), the permanent stoma rate (22 vs. 35%), and significantly increased the rate of primary anastomosis (70 vs 54%).

The most clinically relevant finding of the present study is the significantly lower 1-year stoma rate in patients treated with SEMS as a bridge to surgery, which was the primary outcome of the CROSCO-1 study. While randomized controlled trials and meta-analyses have already established the efficacy of SEMS as a bridge to surgery, real-world prospective data remain limited. The present study complements existing evidence by providing multicenter data from routine clinical practice, reflecting variability in access to SEMS and endoscopic expertise across centers, and including patient-centered outcomes such as quality of life and timing of adjuvant chemotherapy. Although stoma reversal rates did not differ significantly between groups after adjustment, a substantial proportion of patients undergoing emergency surgery were still living with a stoma at one year, confirming the well-known issue of “temporary” stomas becoming permanent after Hartmann’s procedure. In fact, at 1 year following the acute presentation necessitating emergency surgery, more than half of the patients (73.4%) were still living with a stoma, whereas this figure dropped 44.4% among patients treated with SEMS as a bridge to surgery. Furthermore, by converting an emergency into an elective setting, SEMS placement appears to use minimally invasive surgery (laparoscopic and robotic) and to facilitate primary anastomosis without diversion and, when a stoma is required, improves the likelihood of successful reversal.

The study sample size was calculated based on retrospective studies available at the time the protocol was developed [1, 14]. The relatively small number of patients undergoing SEMS (*n* = 72) likely reflects the limited availability of operative endoscopy and the requirement for specific expertise in SEMS placement. Importantly, the procedure failure rate was low (8.3%) compared with the 13% reported in the literature [15], and no perforations were observed. Given the known association between perforation and local recurrence, these findings suggest that SEMS placement was performed in experienced centers. The presence of a stoma has a considerable impact on the daily lives of our patients. Quality of life represents a key outcome from the patient’s perspective and is often underreported in studies on obstructive colorectal cancer. In the most recent literature, only two single-center retrospective studies [16, 17] have specifically evaluated the impact on quality of life in patients with obstructive left-sided colon cancer requiring urgent surgical management. In both studies, the use of SEMS as a bridge to surgery, through its association with reduced stoma formation, was shown to confer significant benefits in terms of social functioning, personal autonomy, and psychological well-being. In our study, using the EQ-5D-5L instrument, we observed significantly better outcomes across all five domains in patients managed with a staged approach. Differences were particularly pronounced in self-care, usual activities, pain/discomfort, and anxiety/depression, likely reflecting the combined impact of stoma avoidance, faster recovery, and elective minimally invasive surgery. These findings reinforce the concept that treatment success in this setting should not be assessed solely in terms of morbidity and survival, but also in relation to long-term functional and psychosocial outcomes.

The staged approach was not associated with increased postoperative morbidity or mortality. Major postoperative complications (Clavien–Dindo ≥ III) were comparable between groups, and overall mortality remained low. However, patients in the ESG showed higher rates of hospital readmission (15.9%) and reoperation (4.3%) within 30 days. These findings should be interpreted in light of potential selection bias, as patients undergoing emergency surgery were generally older and had a higher burden of comorbidities. This observation adds a novel dimension to the existing literature. While early and 90-day postoperative morbidity and mortality were comparable between the two strategies, consistent with previous trials [18, 19] and meta-analyses [20], the higher rate of 30-day readmission highlights the critical role of patient condition at the time of acute obstruction. Bridging with SEMS, by allowing clinical stabilization and optimization, may therefore facilitate a safer and more controlled elective resection.

Oncological outcomes in patients with obstructive left-sided colon cancer remain a matter of ongoing debate. In our study, 1-year rates of distant metastases and local recurrence were comparable between the two strategies. However, given the limited duration of follow-up, these findings should be considered preliminary, and no definitive conclusions regarding oncologic safety can be drawn. Although long-term oncologic outcomes were not the primary focus of the present analysis and will be addressed in a subsequent study, our results are consistent with previous trials [21, 22] and propensity-matched analyses suggesting that, in experienced centers and carefully selected patients, SEMS does not adversely affect oncologic outcomes.

A further clinically meaningful advantage observed in the SEMS group was the shorter time to initiation of adjuvant chemotherapy, along with a higher proportion of patients starting systemic treatment within 90 days. This finding represents a novel contribution compared with previously published studies. Delays in adjuvant chemotherapy are known to negatively impact oncological outcomes in stage II–III colon cancer. Emergency surgery, postoperative complications, and prolonged recovery associated with stoma management may all contribute to treatment delays. By reducing surgical trauma and postoperative burden, SEMS as a bridge to surgery may indirectly enhance adherence to oncological treatment pathways.

This study has several important strengths that deserve consideration. First, CROSCO-1 represents one of the largest national, multicenter prospective cohort studies specifically addressing the management of acute left-sided malignant colonic obstruction in a real-world setting. The prospective design and inclusion of consecutive patients across multiple centers enhance the robustness of the data and minimize selection bias typically associated with retrospective series. Second, the study captures contemporary clinical practice, reflecting the actual availability of endoscopic expertise and surgical strategies, thereby increasing the external validity and generalizability of the findings. Third, the comprehensive assessment of outcomes—including stoma rate, timing of adjuvant chemotherapy initiation, and patient-reported quality of life—provides a holistic evaluation of treatment strategies beyond traditional morbidity and mortality metrics. In particular, the inclusion of validated quality-of-life measures offers valuable patient-centered insights that are often underrepresented in this clinical scenario. Finally, the absence of stent-related perforations and the low procedural failure rate highlight the relevance of expertise and centralization, reinforcing the applicability of SEMS as a bridge to surgery when performed in experienced centers. 

However, the study has some limitations. First, its observational and non-randomized design precludes any causal inference regarding the effect of treatment strategy on outcomes. Treatment allocation was based on local expertise and availability of endoscopic services, introducing the potential for selection bias and residual confounding. This may have influenced both patient selection and outcomes, as patients undergoing SEMS were more likely to be treated in centers with specific expertise and resources. Although multivariable regression was used to adjust for potential confounders, more advanced approaches such as propensity score matching were not performed due to sample size limitations, particularly in the SEMS group, which could have resulted in a significant loss of statistical power. Therefore, residual confounding cannot be excluded. However, this approach reflects real-world national clinical practice, capturing the variability in access to SEMS and local expertise across centers, and therefore enhances the external validity of the findings. Nevertheless, SEMS placement was predominantly performed in centers with specific endoscopic expertise, which may limit generalizability to lower-volume settings. Finally, the follow-up is currently limited to one year, precluding definitive conclusions on long-term oncological safety. Ongoing follow-up and the planned second phase of the study (CROSCO-2), specifically designed to assess long-term oncological outcomes, will be essential to address this limitation.

## Conclusion

The CROSCO-1 study suggests that SEMS as a bridge to surgery may represent a safe and feasible option for selected patients with obstructive left-sided colon cancer. In this cohort, its use was associated with a lower 1-year stoma rate compared with emergency surgery, along with potential benefits in terms of quality of life, feasibility of a minimally invasive approach, and continuity of oncological care, without an apparent increase in short-term morbidity. However, given the observational design of the study, these findings should be interpreted as associative rather than causal, and residual confounding cannot be excluded.

These results support a tailored, multidisciplinary approach in experienced centers, where SEMS as a bridge to surgery may be considered a reasonable alternative to emergency surgery in appropriately selected patients.

Further follow-up of this cohort will be essential to better define long-term oncological outcomes.

## Supplementary Information

Below is the link to the electronic supplementary material.

Supplementary file1 (DOCX 21 KB)

Supplementary file2 (DOCX 22 KB)

Supplementary file3 (DOCX 15 KB)

## Data Availability

Authors state that the work described has not been published previously, that it is not under consideration for publication elsewhere, and that its publication is approved by all authors.
