# Correction: Colonic resection or self-expanding metal stents for obstructive left colon cancer: results of a national multicenter prospective cohort study (CROSCO-1)

**DOI:** 10.1007/s00464-026-13062-3

**Published:** 2026-07-24

**Authors:** Alessio Giordano, Manuela Mastronardi, Paola Fugazzola, Giulia Montori, Ferdinando Agresta, Jacopo Martellucci, Fausto Catena, Federico Coccolini, Gabriele Anania, Gianluca Costa, Vincenzo Bottino, Nicola Cillara, Paolo Prosperi, Carlo Bergamini, Mauro Podda, The CROSCO-1 Collaborative Group, Marco Scatizzi, Luigi Ricciardielli, Diego Cuccurullo, Mario Guerrieri, Alberto Sartori, Massimo Sartelli, Luca Ansaloni, Nereo Vettoretto, Monica Ortenzi, Emanuele Botteri, Jacopo Martellucci, Mauro Marzano, Biagio Casagranda, Silvia Palmisano, Alan Biloslavo, Letizia Cecchini, Nicolò de Manzini, Pietro Fransvea, Silvia Tedesco, Caterina Puccioni, Ruggero Bollino, Massimiliano Casadei, Denise Marchesini, Mauro Podda, Lisa Seu, Federica Frongia, Anna Guariniello, Nicola Albertini, Claudia Di Leo, Andrea Borasi, Andrea Barberis, Francesco Ghiglione, Michele De Capua, Maddalena Tarricone, Mauro Ghirardi, Leo Licari, Gianluca Gambino, Vincenzo Sorce, Andrea Celotti, Martina Bonafede, Luca Mattia Quarti, Marco Clementi, Danilo Meloni, Irene Tucceri Cimini, Giorgio Ammerata, Michele Mazza, Giuseppe Sena, Giuseppe Palomba, Giovanni Aprea, Ciro de Martino, Giuseppe Miranda, Enrico Erdas, Gian Luigi Canu, Federico Cappellacci, Manish Kumar Agrawal, Akshay Anand Abhinav, Arun Sonkar, Amit Gupta, Deepak Rajput, Avijit Mondal, Jacopo Andreuccetti, Giusto Pignata, Claudia Zaghi, Franco Poli, Houshang Kalamian, Marco Milone, Anna D’amore, Giovanni De Palma, Giuseppe Curro, Michele Ammendola, Silvia Curcio, Andrea-Pierre Luzzi, Salvatore Carrabetta, Raquel Diaz, Valerio Caracino, Paola Salusso, Diletta Frazzini, Paolo Di Mattia, Chiara Toscano, Debora Maria Di Dio, Valeria Tonini, Lodovico Sartarelli, Maurizio Cervellera, Giuseppe Evola, Luigi Piazza, Lorenzo Ripamonti, Nicolò Tamini, Giulia De Carlo, Francesco Feroci, Carla Vaccaro, Benedetta Pesi, Antonello Mirabella, Stefano Mandalà, Dario Iadicola, Pasquale Cianci, Ivana Conversano, Fabio Curci, Paolo Ialongo, Pisicchio Simona, Rrozhani Valentina, Angelo Benevento, Maria Francesca Chiappetta, Federica Galli, Luigi Boccia, Giacomo Brentegani, Roberto Dusi, Raffaele Galleano, Omar Ghazouani, Elena Gambino, Massimo Calistri, Claudia Paolini, Luca Domenico Bonomo, Chiara Marafante, Antonella Nicotera, Paolo Pietro Bianchi, Giampaolo Formisano, Adelona Salaj, Fabio Staderini, Laura Fortuna, Damiano Bisogni, Rita Laforgia, Angela Pezzolla, Pierluigi Lobascio, Andrea Coratti, Giuseppe Giuliani, Francesco Guerra, Michele Marini, Costanzo Antonio, Michela Caprioli, Christian Cotsoglou, Beatrice Torre, Stefano Granieri

**Affiliations:** 1 Emergency Surgery Unit, Careggi University Hospital, Largo Brambilla 3, 50134 Florence, Italy; 2 General Surgery Unit, Department of Medicine, Surgery and Health Sciences, University Hospital of Trieste, Trieste, Italy; 3 Department of General Surgery I, University of Pavia, IRCCS San Matteo Hospital, Pavia, Italy; 4 Department of General Surgery, Ulss2 Marca Trevigiana, Vittorio Veneto, Treviso Italy; 5 Emergency and Trauma Surgery Unit, Bufalini Hospital, Cesena, Italy; 6 Emergency and General Surgery Unit, Pisa University Hospital, Pisa, Italy; 7 Department of Surgery, Sant’Anna University Hospital, Ferrara, Italy; 8 Colo-Rectal Surgery Unit, Link Campus University, Rome, Italy; 9 Department of General and Laparoscopic Surgery, Bariatric and Metabolic Surgery Unit, Ospedale Evangelico Betania, Naples, Italy; 10 General Surgery Unit, P.O. Santissima Trinità, Cagliari, Italy; 11 Department of Surgical Science, Cagliari State University, Cagliari, Italy

**Correction to: Surgical Endoscopy** 10.1007/s00464-026-12929-9

The original online version of this article was revised to index the names of all members of the CROSCO-1 Collaborative Group.

The original article has been corrected.

