# Supplementary material for: Colonic resection or self-expanding metal stents for obstructive left colon cancer: results of a national multicenter prospective cohort study (CROSCO-1)
Source: Surg Endosc. 2026 Jun 3;40(8):6567–76. doi: 10.1007/s00464-026-12929-9 (PMC13457328; doi:10.1007/s00464-026-12929-9)
Supplement: Supplementary file 1 [file 464_2026_12929_MOESM1_ESM.docx]

**Supplementary Table 1**. Short- and mid-term follow-up outcomes according to treatment group

|  | **ESG (n = 30)** | **SEMS/Bts (n = 10)** | **p value** |
| --- | --- | --- | --- |
| **Complication after stoma reversal** |  |  | 0.480 |
| Adhesion | 2 (6.7%) | 0 (0.0%) |  |
| Anastomotic leak | 1 (3.3%) | 0 (0.0%) |  |
| Intestinal obstruction | 1 (3.3%) | 1 (10.0%) |  |
| Skin infection | 8 (26.7%) | 2 (20.0%) |  |
|  | **ESG (n = 80)** | SEMS/BtS **(n = 8)** | **p value** |
| **Complications at 1 year (ostomy in situ)** | 25 (31.3%) | 2 (25.0%) | 1.000 |
| **Complication type** |  |  |  |
| Incisional hernia | 18 (72%) | 2 (100%) |  |
| Other | 7 (28%) | 0 |  |
|  | **ESG (n = 78)** | SEMS/BtS **(n = 51)** | **p value** |
| **Type of chemotherapy started** |  |  | 0.490 |
| Capecitabine | 12 (15.4%) | 10 (19.6%) |  |
| xelox/folfox | 65 (83.3%) | 39 (76.5%) |  |
| folfirinox±bevacizumab | 1 (1.3%) | 2 (3.9%) |  |
|  | **ESG (n = 73)** | SEMS/BtS **(n = 54)** | **p value** |
| **Type of chemotherapy started - 90 days** |  |  | 0.486 |
| capecitabine | 11 (15.1%) | 11 (20.4%) |  |
| xelox/folfox | 61 (83.6%) | 41 (75.9%) |  |
| folfirinox±bevacizumab | 1 (1.4%) | 2 (3.7%) |  |

Data are presented as number (percentage). EGS: emergency surgery group; SEMS/Bts: self-expanding metal stents (SEMS) as a bridge to surgery (BtS)
