# Supplementary material for: Colonic resection or self-expanding metal stents for obstructive left colon cancer: results of a national multicenter prospective cohort study (CROSCO-1)
Source: Surg Endosc. 2026 Jun 3;40(8):6567–76. doi: 10.1007/s00464-026-12929-9 (PMC13457328; doi:10.1007/s00464-026-12929-9)
Supplement: Supplementary file 2 [file 464_2026_12929_MOESM2_ESM.docx]

**Supplementary Table 2**. Health-related quality of life according to EQ-5D domains by treatment group

| **Mobility** |  |  |  |
| --- | --- | --- | --- |
| **Response level** | **ESG (n = 134)** | **SEMS/BtS (n = 70)** | **p value** |
| Unable to walk about | 3 (2.2%) | 2 (2.9%) | 0.049 |
| Moderate problems | 15 (11.2%) | 3 (4.3%) |  |
| No problems | 78 (58.2%) | 55 (78.6%) |  |
| Severe problems | 1 (0.7%) | 0 (0.0%) |  |
| Slight problems | 37 (27.6%) | 10 (14.3%) |  |
| **Self-care** |  |  |  |
| **Response level** | **ESG (n = 134)** | **SEMS/BtS (n = 70)** | **p value** |
| Unable to wash/dress | 4 (3.0%) | 2 (2.9%) | <0.001 |
| Moderate problems | 20 (14.9%) | 1 (1.4%) |  |
| No problems | 71 (53.0%) | 58 (82.9%) |  |
| Severe problems | 3 (2.2%) | 0 (0.0%) |  |
| Slight problems | 36 (26.9%) | 9 (12.9%) |  |
| **Usual activities** |  |  |  |
| **Response level** | **ESG (n = 134)** | **SEMS/BtS (n = 70)** | **p value** |
| Unable to do usual activities | 3 (2.2%) | 2 (2.9%) | <0.001 |
| Moderate problems | 29 (21.6%) | 2 (2.9%) |  |
| No problems | 55 (41.0%) | 55 (78.6%) |  |
| Severe problems | 2 (1.5%) | 0 (0.0%) |  |
| Slight problems | 45 (33.6%) | 11 (15.7%) |  |
| **Pain/discomfort** |  |  |  |
| **Response level** | **ESG (n = 134)** | **SEMS/BtS (n = 70)** | **p value** |
| Extreme pain/discomfort | 1 (0.7%) | 0 (0.0%) | 0.003 |
| Moderate pain/discomfort | 27 (20.1%) | 7 (10.0%) |  |
| No pain/discomfort | 53 (39.6%) | 48 (68.6%) |  |
| Severe pain/discomfort | 1 (0.7%) | 0 (0.0%) |  |
| Slight pain/discomfort | 52 (38.8%) | 15 (21.4%) |  |
| **Anxiety/depression** |  |  |  |
| **Response level** | **ESG (n = 134)** | **SEMS/BtS (n = 70)** | **p value** |
| Extremely anxious/depressed | 1 (0.7%) | 0 (0.0%) | <0.001 |
| Moderately anxious/depressed | 37 (27.6%) | 6 (8.6%) |  |
| Not anxious/depressed | 35 (26.1%) | 44 (62.9%) |  |
| Severely anxious/depressed | 5 (3.7%) | 1 (1.4%) |  |
| Slightly anxious/depressed | 56 (41.8%) | 19 (27.1%) |  |

Data are presented as number (percentage). EGS: emergency surgery group; SEMS/Bts: self-expanding metal stents (SEMS) as a bridge to surgery (BtS)
