# Supplementary material for: Colonic resection or self-expanding metal stents for obstructive left colon cancer: results of a national multicenter prospective cohort study (CROSCO-1)
Source: Surg Endosc. 2026 Jun 3;40(8):6567–76. doi: 10.1007/s00464-026-12929-9 (PMC13457328; doi:10.1007/s00464-026-12929-9)
Supplement: Supplementary file 3 [file 464_2026_12929_MOESM3_ESM.docx]

**Supplementary Table 3**. Univariate logistic regression for stoma presence at 1 year

| **Predictor** | **OR** | **95% CI** | **p value** |
| --- | --- | --- | --- |
| **Surgical (vs endoscopic)** | 3.448 | 1.241–9.583 | 0.018 |
| **Age (per 1-year increase)** | 1.043 | 1.013–1.073 | 0.004 |
| **Male sex (vs female)** | 1.029 | 0.483–2.192 | 0.942 |
| **CCI (per 1-point increase)** | 1.185 | 1.022–1.374 | 0.025 |
| **BMI (per 1 kg/m² increase)** | 1.044 | 0.951–1.146 | 0.366 |
| **ASA grade (overall)** | — | — | 0.181 |
| **└ ASA I (vs ASA IV)*** | 0.125 | 0.014–1.098 | 0.061 |
| **└ ASA II (vs ASA IV)*** | 0.200 | 0.040–0.996 | 0.049 |
| **└ ASA III (vs ASA IV)*** | 0.296 | 0.062–1.416 | 0.127 |
| **Postoperative complications (yes vs no)** | 0.877 | 0.348–2.209 | 0.781 |

Data are presented as number (percentage). EGS: emergency surgery group; SEMS/Bts: self-expanding metal stents (SEMS) as a bridge to surgery (BtS), BMI: Body Mass Index; CCI: Charlson Comorbidity Index; ASA score: American Society of Anesthesiologists score
